# Skills of endemic disease control agents in the technical training of the Health with Agent Program: a descriptive study, Brazil, 2023

**DOI:** 10.1590/S2237-96222026v35e20250563.en

**Published:** 2026-07-20

**Authors:** Bruna Lazzeri, Diogo Pilger, Carmen Lúcia Mottin Duro, Marilise Oliveira Mesquita, Fabiana Schneider Pires, Daniela Riva Knauth, Luciana Barcellos Teixeira

**Affiliations:** 1 Universidade Federal do Rio Grande do Sul Porto Alegre Porto Alegre RS Brazil Universidade Federal do Rio Grande do Sul, Porto Alegre, RS, Brazil

**Keywords:** Endemic Disease Control Agents, Public Health Surveillance, Professional Training, Health Education, Environmental Health Surveillance, Agente de control de endemias, Vigilancia en salud pública, Capacitación profesional, Educación en salud, Vigilancia en salud ambiental

## Abstract

**Objective:**

To describe the knowledge and self-perceived skills of endemic disease control agents before the specific technical training of the Health with Agent Program (PSA).

**Method:**

Descriptive study using data from students of the Health with Agent Program, collected through a self-administered online questionnaire. The instrument included sociodemographic characterization and self-perception of skills in four domains: (a) education, planning, and actions within the community, (b) environmental surveillance, (c) epidemiological surveillance, and (d) health surveillance and two dimensions: (1) needs recognition and action planning, and (2) execution, with responses on a Likert scale. Data were analyzed using R statistical software (version 4.4.1) and presented as absolute and relative frequencies.

**Results:**

The sample included 3,730 endemic disease control agents, of whom 51.8% were women, 67.0% were Black, 49.7% had completed high school, and 67.9% had direct formal affiliation with the municipality. Of the total, 90.6% worked in urban areas, and 39.7% were from the Northeast region. Self-perceived competence ranged from 47.0% to 94.0%. The highest self-perceived proficiencies were observed in educational activities on endemic disease prevention (90.3%), pesticide application (90.5%), and vector control (94.0%). The lowest levels were observed in environmental surveillance, particularly regarding damage from climate events and the utilization of organic waste, with more than 50.0% of participants reporting that they did not know how to perform the tasks, had limited knowledge, or had doubts.

**Conclusion:**

The results indicate that, although endemic disease control agents perceive themselves as competent in traditional endemic control initiatives, there are gaps in emerging areas such as environmental surveillance.

## Resumen

**Objetivo:**

Describir los conocimientos y las habilidades autopercibidas por los agentes de control de endemias antes de la formación técnica específica del Programa Salud con Agente.

**Método:**

Estudio descriptivo con datos de estudiantes del Programa Salud con Agente, recolectados mediante un cuestionario en línea autoadministrado. El instrumento incluyó la caracterización sociodemográfica y la autopercepción de habilidades en cuatro dominios: (a) educación, planificación y acciones comunitarias; (b) vigilancia ambiental; (c) vigilancia epidemiológica; y (d) vigilancia sanitaria, y en dos dimensiones: (1) reconocimiento de necesidades y planificación de acciones; y (2) ejecución, con respuestas en escala Likert. Los datos fueron analizados en el software estadístico R (versión 4.4.1) y se presentaron en frecuencias absolutas y relativas.

**Resultados:**

La muestra incluyó 3.730 agentes de control de endemias, de los cuales el 51,8% eran mujeres, 67,0% se identificaron como personas negras, 49,7% tenían educación secundaria completa y 67,9% tenían vínculo laboral directo con el municipio. Del total, 90,6% trabajaban en áreas urbanas y 39,7% en la región Nordeste. La autopercepción de competencia varió entre 47,0% y 94,0%. Las mayores competencias autopercibidas se observaron en actividades educativas sobre la prevención de enfermedades endémicas (90,3%), la aplicación de pesticidas (90,5%) y el control de vectores (94,0%). Las menores se concentraron en el área de vigilancia ambiental, especialmente respecto a los daños ocasionados por eventos climáticos y al aprovechamiento de residuos orgánicos, superando el 50,0% al considerar a los participantes que declararon no saber realizar las acciones, saber poco o tener dudas.

**Conclusión:**

Los resultados indican que, aunque los agentes de control de endemias se perciban competentes en las acciones tradicionales de control de endemias, existen brechas en áreas emergentes, como la vigilancia ambiental.

**Ethical aspects**

This research respected ethical principles, having obtained the following approval data:

Research ethics committee Universidade Federal do Rio Grande do Sul

Opinion number 5,679,570

Approval date 60867922.6.0000.5347

Certificate of submission for ethical appraisal 04/2023

Informed consent form Obtained from all participants prior to data collection.

## Introduction

Endemic disease control agents (*Agentes de Combate às Endemias*, ACE) play a fundamental role in disease surveillance and control in Brazil, acting in communities to identify and reduce health risks. Their responsibilities include entomological and malacological research, environmental management, monitoring of environmental factors, and educational and community mobilization activities. In addition to technical expertise in vector control, endemic disease control agents need to work in an integrated manner with primary health care and understand the social determinants of health (1).

Significant structural challenges hamper the work of endemic disease control agents. Fragmentation of surveillance activities results in a lack of coordination across sectors, services, and professionals, leading to isolated and poorly coordinated practices. This compromises the effectiveness of interventions due to difficulties integrating with primary health care, unclear or overlapping responsibilities, and the absence of effective communication flows for joint planning (2–5). 

Training for endemic disease control agents has focused on operational training, particularly in identifying and eliminating disease-vector breeding sites, such as those associated with dengue, malaria, and leishmaniasis (3,6). Although this approach has contributed to outbreak reduction, its limited scope restricts the role of endemic disease control agents in broader collective health strategies (5). The lack of effective integration between endemic disease control agents and primary health care teams compromises the effectiveness of public health interventions.

The shared responsibilities between endemic disease control agents and community health agents were established in the 2017 national primary health care policy (7) and reinforced by the technical guide on the integration of health surveillance and primary health care (8). Shared responsibilities include developing health promotion and disease prevention activities and providing community guidance on symptoms, risks, and disease vectors. Agents also play a key role in informing and mobilizing the community to implement simple environmental management measures and other environmental interventions beyond vector control (7,8).

As part of the strengthening and qualification of the Brazilian Unified Health System (*Sistema Único de Saúde*, SUS), the Health with Agent Program (*Programa Saúde com Agente*, PSA) was established by the Ordinance GM/MS No. 3,241/2020, aimed at the specialized training of endemic disease control agents and community health agents (9). It was the first technical course offered by the Ministry of Health (*Ministério da Saúde*, MS) with positions distributed nationwide, aligned with the national primary health care policy and, more recently, with the national directive for integrating these two professional categories (7,1). 

The Health with Agent Program (PSA) is structured with theoretical activities in a virtual learning environment and supervised field practices, promoting the articulation between technical knowledge and local context. The theoretical stage is divided into two parts: the first covers topics common to both endemic disease control agents and community health agents, and the second focuses on area-specific knowledge. This training encourages the development of essential competencies in environmental, epidemiological, and health surveillance (10).

In the context of the Health with Agent Program (PSA), this descriptive study aimed to present the self-perceived knowledge and skills of endemic disease control agents before the specific technical training. The study helps identify knowledge gaps and supports the improvement of professional qualifications, in alignment with the guidelines for continuing health education. The results may inform more effective, context-specific educational strategies, thereby strengthening public health surveillance policies in Brazil.

## Methods

### Study design

This study is part of the research project entitled “Training in the Health with Agent Program and its impact on community health.” The objective was to describe the self-perceived skills of students enrolled in the technical course in health surveillance, prior to the specific training intended exclusively for endemic disease control agents, to identify training gaps to support the technical qualification of these professional categories, and to inform curricular adjustments. 

### Setting

The study was conducted within the scope of the Health with Agent Program (PSA), an initiative of the Ministry of Health (MS) aimed at the technical training of endemic disease control agents and community health agents nationwide. The course is structured in two stages: the first, common to both professional categories, and the second, specific to each category. 

### Participants

This study included exclusively endemic disease control agents enrolled in the technical course in health surveillance with an emphasis on endemic disease control (42,976 individuals). Participation was voluntary and preceded by acceptance of the informed consent form in digital format, agreement to which was a prerequisite for access to the questionnaire on self-perceived skills related to work as an endemic disease control agent.

Data were collected via a self-administered online questionnaire sent by email to students in the sixth month of the course, with responses accepted from 8 February 2023 to 28 March 2023.

### Variables

The following sociodemographic variables were assessed: sex (female, male, and prefer not to disclose/other), race/skin color (categorized according to the Brazilian Institute of Geography and Statistics (*Instituto Brasileiro de Geografia e Estatística*, IBGE) as: Brown (Brazilian mixed race), Black, White, Asian, Indigenous, and prefer not to disclose), age (in years, categorized as: 20–29, 30–39, 40–49, 50–59, and ≥60 years), educational level (incomplete high school, complete high school, incomplete higher education, complete higher education, incomplete postgraduate education, and complete postgraduate education), macroregions (Northeast, Southeast, North, Central-West, and South), work setting (urban area, rural are, riverside community, Indigenous community, and other), length of service as an endemic disease control agent (in years, categorized as: 0–2, 3–5, 6–10, 11–15, 16–20, and >20 years), and work affiliation (direct formal affiliation with the municipality, direct formal affiliation with the state, outsourcing contract, do not know/prefer not to disclose, and other). 

The skills of endemic disease control agents were investigated through four main domains of the self-perceived skills questionnaire: (a) education, planning, and activities within the community, (b) environmental surveillance, (c) epidemiological surveillance, and (d) health surveillance, subdivided into two dimensions: (1) recognition of needs and action planning and (2) execution of activities. A total of 25 items from the self-perceived skills questionnaire were used, with responses on a five-point Likert scale (“do not know how to do it,” “know a little how to do it,” “have some doubts,” “know how to do it,” and “know very well how to do it”). 

### Data sources and measurement

Data were collected using a structured questionnaire developed specifically for this study, comprising two main sections: sociodemographic profile and self-perceived skills of endemic disease control agents. The instrument did not undergo a full psychometric validation process; however, its development followed a rigorous methodology, divided into four stages, described below:

Stage 1: bibliographic review conducted in October 2022 in the Virtual Health Library (*Biblioteca Virtual em Saúde*, BVS), including publications from 2018 to 2022 and documents such as pedagogical projects, national primary health care policy guidelines, and legislation relevant to the professional responsibilities of endemic disease control agents.

Stage 2: construction of the instrument, organized into six domains: education, planning, and actions within the community; work process; environmental surveillance within the community; epidemiological surveillance within the community; health surveillance within the community; and first aid. These domains encompass the technical competencies required, understood in this study as the manifestation of “know-how,” corresponding to the ability to mobilize cognitive, socio-affective, and psychomotor resources to address complex situations (11). Each skill was assessed according to two dimensions, termed “recognition of needs and action planning” and “execution,” using a five-point Likert scale: “do not know how to do it,” “know a little how to do it,” “have some doubts,” “know how to do it,” and “know very well how to do it”.

Stage 3: content and semantic structure validation conducted by eight specialists holding doctoral degrees and with experience in the field, in accordance with Lynn’s methodological recommendation, which establishes ideal participation between five and ten experts.

Stage 4: *face validity* assessment conducted with 15 professionals, randomly selected from among endemic disease control agents and community health agents. All participants had at least 6 months of professional experience and were actively employed in their positions. The objective was to assess clarity, comprehension, and relevance, and to collect suggestions for improving the questionnaire. 

The final instrument comprised 47 items distributed across six domains. For this study, 25 items belonging to four of these domains were selected (described in the subsection “Variables”).

### Bias

Due to online data collection and the self-administered nature of the questionnaire, selection and information biases may be present; these are discussed in the Discussion section, which addresses the study limitations. Measures were implemented to minimize bias, including excluding duplicate responses and refusals.

### Study size 

Among enrolled students who accessed the questionnaire during the available period, 51 chose not to participate in the study; 588 duplicate responses were identified and excluded; and 440 participants did not fully complete the skills assessment questions. The final sample comprised 3,730 participants. No probabilistic sampling was performed, and a convenience sample was constituted within the eligible population.

### Quantitative variables

Continuous quantitative variables, such as age and length of service, were categorized into predefined ranges to facilitate analysis and minimize distortions from extreme values. The categories were established based on intervals relevant to participant characterization and were also consistent with the available literature.

### Statistical methods

Data organization and cleaning were performed using Microsoft Excel. Statistical analyses were conducted using R software, version 4.4.1. Absolute and relative frequencies were calculated for categorical variables and for the domains and dimensions of the assessed skills. Only complete responses were considered valid. 

## Results

Of the 3,730 participating endemic disease control agents, 51.8% were female. The Black race/skin color group (Black and Brown) predominated, accounting for 67.0% of the sample. Nearly half of the sample (49.1%) had completed or were in the process of completing higher education, including postgraduate degrees. Most participants (90.6%) worked in urban areas, and 39.7% were concentrated in the Northeast region. A total of 67.9% had a direct formal affiliation with the municipality. The predominant age group was 40–49 years (39.9%). Regarding length of service, 25.7% had 11–15 years of work experience (Table 1).

**Table 1:** Sociodemographic characteristics of the sample of endemic disease control agents participating in the Health with Agent Program (*Programa Saúde com Agente*, PSA). Brazil, 2023 (n=3,730)

Characteristics	n (%)
**Sex **	
Female	1,934 (51.8)
Male	1,778 (47.7)
Prefer not to disclose/Other	18 (0.5)
**Race/skin color**	
Brown (Brazilian mixed race)	1,986 (53.2)
White	1,128 (30.2)
Black	514 (13.8)
Asian	44 (1.2)
Indigenous	31 (0.8)
Prefer not to disclose	27 (0.7)
**Age **(years)	
20–29	243 (6.5)
30–39	1,161 (31.1)
40–49	1,489 (39.9)
50–59	693 (18.6)
≥60	144 (3.9)
**Educational level**	
Incomplete high school	50 (1.3)
Complete high school	1,852 (49.7)
Incomplete higher education	522 (14.0)
Complete higher education	808 (21.7)
Incomplete postgraduate education	93 (2.5)
Complete postgraduate education	405 (10.9)
Macroregions	
Northeast	1,480 (39.7)
Southeast	1,126 (30.2)
North	434 (11.6)
Central-West	346 (9.3)
South	344 (9.2)
**Work setting**	
Urban area	3,378 (90.6)
Rural area	243 (6.5)
Riverside community	15 (0.4)
Indigenous community	8 (0.2)
Other	86 (2.3)
**Length of service **(years)	
0–2	374 (10.0)
3–5	477 (12.8)
6–10	807 (21.6)
11–15	959 (25.7)
16–20	604 (16.2)
>20	509 (13.7)
**Work affiliation **	
Direct formal affiliation with the municipality	2,533 (67.9)
Direct formal affiliation with the state	100 (2.7)
Outsourcing contract (company, foundation, or social organization)	48 (1.3)
Do not know/Prefer not to disclose	113 (3.0)
Other	936 (25.1)

Between 79.0% and 94.0% of endemic disease control agents reported that they “know how to do” or “know how to do very well” the proposed activities, with the highest percentages observed for educational activities on endemic disease prevention (90.3%), vector control (94.0%), and pesticide application (90.5%) (Tables 2, 4, and 5).

**Table 2:** Education, planning, and territorial action skills prior to the specific technical training of endemic disease control agents in the Health with Agent Program (*Programa Saúde com Agente*, PSA). Brazil, 2023 (n=3,730)

Education, planning, and community-based actions	Do not know how to do it n (%)	Know a little how to do it n (%)	Have some doubts n (%)	Know how to do it n (%)	Know very well how to do it n (%)	
**Recognition of needs and action planning**						
Plan with the community educational actions for health promotion.	52 (1.4)	569 (15.3)	171 (4.6)	2,116 (56.7)	822 (22.0)	
Plan together with the community actions to combat environmental damage.	112 (3.0)	643 (17.2)	436 (11.7)	1,929 (51.7)	610 (16.4)	
Identify situations of social risk within the community (situations of violence, social vulnerability among others).	56 (1.5)	406 (10.9)	308 (8.2)	2,085 (55.9)	875 (23.5)	
Implementation						
Mobilize the community to participate in educational activities.	60 (1.6)	416 (11.1)	178 (4.8)	2,058 (55.2)	1,018 (27.3)	
Carry out education actions on the Prevention of endemic diseases.	26 (0.7)	235 (6.3)	101 (2.7)	1,949 (52.3)	1,419 (38.0)	
Carry out actions to combat environmental damage.	109 (2.9)	574 (15.4)	448 (12.0)	1,890 (50.7)	709 (19.0)	

Environmental surveillance activities showed the highest percentages of agents who reported not knowing how to perform the actions, ranging from 9.5% to 13.8% for activities related to damage caused by heavy rainfall, drought periods, air quality, and organic waste utilization (Table 3). When agents who reported knowing how to perform little or who had doubts were included, the percentage for these activities increased to 53.0% (Table 3). 

**Table 3:** Environmental surveillance skills within the community, before the technical training of agents to combat endemics in the Health with Agent Program (*Programa Saúde com Agente*, PSA). Brazil, 2023 (n=3,730)

Environmental surveillance at the local level	Do not know how to do it n (%)	Know a little how to do it n (%)	Have some doubts n (%)	Know how to do it n (%)	Know very well how to do it n (%)
**Recognition of needs and action planning**					
Plan actions to address environmental damage (caused by droughts, heavy rainfall, floods, among others).	514 (13.8)	748 (20.1)	713 (19.1)	1,326 (35.5)	429 (11.5)
Plan actions to collect cups, bottles, glass, and plastics for recycling.	70 (1.9)	318 (8.5)	126 (3.4)	1,866 (50.0)	1,350 (36.2)
Plan actions for waste recovery and composting.	446 (11.9)	716 (19.2)	629 (16.9)	1,429 (38.3)	510 (13.7)
Implementation					
Carry out community-based actions to reduce environmental pollution (air, water, and noise).	353 (9.5)	727 (19.5)	530 (14.2)	1,632 (43.7)	488 (13.1)
Carry out community-based actions to reduce environmental damage caused by droughts, heavy rainfall, floods, among others.	415 (11.1)	735 (19.7)	668 (17.9)	1,481 (39.7)	431 (11.6)
Carry out community-based actions to collect cups, bottles, glass, and plastics for recycling.	100 (2.7)	402 (10.8)	175 (4.7)	1,935 (51.9)	1,118 (29.9)
Carry out actions together with the community for organic waste recovery and composting.	441 (11.8)	714 (19.1)	645 (17.3)	1,423 (38.2)	507 (13.6)

Epidemiological surveillance and community-based planning skills showed high levels of self-perceived competence, with 79.1% for action records and 94.0% for control of vector-borne diseases. Indicators related to workers’ health ranged from 58.8% to 61.4% among those who reported knowing how to perform the activities (Table 4). 

**Table 4:** Epidemiological surveillance skills within the community prior to the technical training of endemic disease control agents in the Health with Agent Program (*Programa Saúde com Agente*, PSA). Brazil, 2023 (n=3,730)

Epidemiological surveillance within the community	Do not know how to do it n (%)	Know a little how to do it n (%)	Have some doubts n (%)	Know how to do it n (%)	Know very well how to do it n (%)
**Recognition of needs and action planning**					
Plan with the community actions to prevent endemic diseases.	47 (1.3)	350 (9.4)	150 (4.0)	1,950 (52.3)	1,233 (33.0)
Plan actions to promote workers ‘ health and prevent work-related diseases.	259 (6.9)	618 (16.6)	565 (15.1)	1,658 (44.5)	630 (16.9)
Plan actions that meet the needs and demands of the community.	207 (5.6)	697 (18.7)	498 (13.3)	1,736 (46.5)	592 (15.9)
Implementation					
Stimulate actions to promote workers ‘ health and prevent work-related diseases.	299 (8.0)	654 (17.5)	585 (15.7)	1,591 (42.7)	601 (16.1)
Carry out actions to control vector-borne diseases (dengue, zika, chikungunya, yellow fever among others).	19 (0.5)	145 (3.9)	60 (1.6)	1,520 (40.8)	1,986 (53.2)
Record the data of the epidemiological surveillance actions developed.	143 (3.8)	332 (8.9)	305 (8.2)	1,678 (45.0)	1,272 (34.1)

Health surveillance indicated high rates of self-perceived mastery in identifying and guiding on zoonoses (84.8%), sanitary inspections (82.3%), and pesticide application (90.5%). Regarding improper waste disposal, risk of collapse, and consumption of unsuitable products, between 59.9% and 67.5% of endemic disease control agents reported mastery of the assessed practices. Between 2.5% and 7.8% reported a lack of competence in the activities (Table 5).

**Table 5:** Health surveillance skills within the community, before the technical training of agents to combat endemics in the Health with Agent Program (*Programa Saúde com Agente*, PSA). Brazil, 2023 (n=3,730)

Health surveillance within the community	Do not know how to do it n (%)	Know a little how to do it n (%)	Have some doubts n (%)	Know how to do it n (%)	Know very well how to do it n (%)
**Recognition of needs and action planning**					
Identify problems at the local level (improper waste disposal, landslide risks, among others).	270 (7.2)	567 (15.2)	375 (10.1)	1,730 (46.4)	788 (21.1)
Identify risks for disease transmission from animals.	93 (2.5)	535 (14.3)	286 (7.7)	1,892 (50.7)	924 (24.8)
Plan actions to prevent the community from consuming unsafe products (food, water, among others).	292 (7.8)	649 (17.4)	555 (14.9)	1,696 (45.5)	538 (14.4)
Implementation					
Carry out sanitary inspections in households, vacant lots, storage areas, and commercial establishments.	149 (4.0)	283 (7.6)	229 (6.1)	1,528 (41.0)	1,541 (41.3)
Provide guidance to the community on animal care (dogs, cats, horses, among others) to prevent disease transmission.	70 (1.9)	323 (8.7)	171 (4.6)	1,799 (48.2)	1,367 (36.6)
Apply pesticides (larvicides and insecticides).	83 (2.2)	149 (4.0)	122 (3.3)	1,511 (40.5)	1,865 (50.0)

## Discussion

Most of the endemic disease control agents investigated in this study reported high confidence in activities related to the prevention and control of endemic diseases, epidemiological surveillance, and pesticide handling. However, gaps were identified in environmental surveillance, particularly in the management of damage from climatic events and in the utilization of organic waste. 

The methodology, based on self-reported questionnaires, although subject to overestimation bias in competencies, proved reliable in identifying areas of mastery and weaknesses, as the internal consistency of the findings supported both indicators of excellence and the difficulties reported (12). 

Although the sample consisted of students enrolled in a technical course with remote classes, online data collection may have limited participation among individuals with limited access to and fluency in digital technologies, potentially underrepresenting more vulnerable groups or those with lower educational attainment (13). 

The study advanced in demographic terms, considering that the literature is predominantly limited to small-scale regional studies (14,15). 

Although this study did not include an objective assessment of skills, it is recognized that each scientific investigation offers a specific perspective on the phenomenon under study, contributing particular elements to a broader understanding of the topic. The findings presented herein provided important evidence regarding the self-perception of endemic disease control agents.

A significant increase in female participation in the profession, which has historically been male-dominated, was observed, corroborating findings from previous studies and trends in other health occupations (14,15). The predominance of individuals aged over 40 years, combined with an average length of service exceeding 10 years, suggested an experienced workforce, a factor that may favor the quality of professional practice (5,14,15). The ongoing need for continuing education strategies aligned with the National Permanent Health. Education Policy (*Política Nacional de Educação Permanente em Saúde*, PNEPS) emphasizes updating knowledge and practices in light of technological advances and emerging demands (16–18). 

Most endemic disease control agents self-identified as having Brown race/skin color, which, together with those who self-identified as Black, composed the contingent of Black individuals within this professional category. This racial composition is consistent with previous studies on the sociodemographic profile of endemic disease control agents and community health agents in Brazil, reflecting the predominant inclusion of Black individuals, particularly in community-based roles (19). 

The predominant educational level observed in this study was consistent with the minimum requirements for the position, and a significant proportion of participants had educational attainment above the minimum, indicating potential for professional qualification and the expansion of technical competencies within the category (14,15,19). The importance of continuing training and professional valorization policies that account for educational heterogeneity and encourage the development of critical and intersectoral skills is highlighted (1,19).

In this study, endemic disease control agents reported mastery of most activities. The lowest levels of perceived mastery were observed in environmental surveillance, particularly regarding environmental damage from heavy rainfall, drought, air quality, and the utilization of organic waste. Lower self-perceived proficiency, including in the field of education, was also observed in the planning and execution of environmental activities. This gap may stem from the historical emphasis on short, informal operational training and from structural challenges within the Brazilian Unified Health System, such as a lack of clarity regarding the responsibilities of endemic disease control agents and challenges in implementing intersectoral actions (20–23). 

Fragmentation between endemic disease control agents and community health agents exacerbates this limitation, compromising the effectiveness of surveillance actions and community adherence. Organizational and procedural barriers, such as the absence of communication flows or integrated protocols, also hinder coordination between primary health care and health surveillance (2,24,25). Overcoming this fragmentation requires articulating competencies within integrated work models that combine generalist and specialized functions, thereby strengthening territorial linkage and responses to the social and environmental determinants of health.

The relationship between health and the environment has been recognized since 1986, following the 8th National Health Conference (8ª Conferência Nacional de Saúde), and was consolidated in the 1988 Constitution and in Law No. 8,080/1990 (26,27), which defines environmental surveillance as a fundamental component of the Brazilian Unified Health System. Despite this, endemic disease control has historically focused on isolated actions, without comprehensively addressing the social and environmental determinants of health (23). The reorganization of the Health and Environment Surveillance Secretariat (*Secretaria de Vigilância em Saúde e Ambiente*, SVSA) (Decree No. 11,358/2023) (28) and the incorporation of endemic disease control agents into primary health care through the 2017 National Primary Health Care Policy represent milestones in expanding the responsibilities of these professionals in environmental surveillance and workers’ health (7).

This study showed that the highest levels of self-perception were concentrated in vector control activities, educational activities, and pesticide handling, reflecting a reactive, campaign-based sanitary model of action (4). This dynamic has been observed in dengue control and in investigations on leprosy and Chagas disease (29,30), suggesting a consistent pattern of siloed practice. To overcome this compartmentalization, it is essential to train endemic disease control agents in the planning and execution of intersectoral actions, thereby promoting the valorization of integrated territorial practices. 

The need to intensify coordination between primary health care and health surveillance through initiatives such as the Health with Agent Program (PSA) is reinforced, with a view toward a more resolutive and participatory Brazilian Unified Health System (SUS). This strategy contributes to the transition from the traditional model of “endemic disease management,” characterized by pesticide use, to a model of “endemic disease control” grounded in territorial actions and population-based environmental education, which requires a long-term process of structural change (1,15,20). 

This study conducted a situational analysis prior to technical training for endemic disease control agents within the Health with Agent Program (PSA), highlighting consolidated competencies and specific training needs. These findings may guide curriculum development and support pedagogical strategies targeted at the identified gaps, contributing to integrated and qualified training within the scope of the Brazilian Unified Health System (SUS).

## Data Availability

The data are part of ongoing research and will be made available by the Ministry of Health in the future in anonymized form.
