# Supplementary material for: Skills of endemic disease control agents in the technical training of the Health with Agent Program: a descriptive study, Brazil, 2023
Source: Epidemiol Serv Saude. 2026 Jul 20;35:e20250563. doi: 10.1590/S2237-96222026v35e20250563.en (PMC13686293; doi:10.1590/S2237-96222026v35e20250563.en)
Supplement: Translation

# Translation: Habilidades dos agentes de combate às endemias na formação técnica do Programa Saúde com Agente: estudo descritivo, Brasil, 2023

**DOI:** 10.1590/S2237-96222026v35e20250563.pt

**Authors:** Bruna Lazzeri, Diogo Pilger, Carmen Lúcia Mottin Duro, Marilise Oliveira Mesquita, Fabiana Schneider Pires, Daniela Riva Knauth, Luciana Barcellos Teixeira

**Affiliations:** 1 Universidade Federal do Rio Grande do Sul Porto Alegre Porto Alegre RS Brasil Universidade Federal do Rio Grande do Sul, Porto Alegre, RS, Brasil

**Keywords:** Agente de Combate às Endemias, Vigilância em Saúde Pública, Capacitação Profissional, Educação em Saúde, Vigilância em Saúde Ambiental

## Abstract

**Objetivo:**

Descrever os conhecimentos e as habilidades autopercebidas pelos agentes de combate às endemias previamente à formação técnica específica do Programa Saúde com Agente.

**Método:**

Estudo descritivo com dados de estudantes do Programa Saúde com Agente coletados por questionário online autopreenchido. O instrumento incluiu caracterização sociodemográfica e autopercepção das habilidades em quatro domínios – (a) educação, planejamento e ações no território, (b) vigilância ambiental, (c) vigilância epidemiológica e (d) vigilância sanitária e duas dimensões: (1) reconhecimento de necessidades e planejamento de ações e (2) execução – com respostas em escala Likert. Os dados foram analisados no software estatístico R versão 4.4.1, apresentados em frequências absolutas e relativas.

**Resultados:**

A amostra incluiu 3.730 agentes de combate às endemias, dos quais 51,8% eram mulheres, 67,0% negros, 49,7% tinham ensino médio completo e 67,9% tinham vínculo direto com o município. Do total, 90,6% atuavam em áreas urbanas, 39,7% eram da região Nordeste. A autopercepção de competência variou de 47,0% a 94,0%. As maiores proficiências autopercebidas foram observadas em ações educativas sobre prevenção de doenças endêmicas (90,3%), aplicação de agrotóxicos (90,5%) e controle de vetores (94,0%). As menores concentraram-se na área de vigilância ambiental, especialmente quanto aos danos decorrentes de eventos climáticos e ao aproveitamento de rejeitos orgânicos, com mais de 50,0% quando considerados os participantes que declararam não saber fazer, saber pouco ou apresentaram dúvidas.

**Conclusão:**

Os resultados indicam que, embora os agentes de combate às endemias percebam-se competentes nas ações tradicionais de controle de endemias, há lacunas em áreas emergentes, como a vigilância ambiental.

**Aspectos éticos**

Esta pesquisa respeitou os princípios éticos, obtendo os seguintes dados de aprovação:

Comitê de ética em pesquisa Universidade Federal do Rio Grande do Sul

Número do parecer 5.679.570

Data de aprovação 3/10/2022

Certificado de apresentação de apreciação ética 60867922.6.0000.5347

Registro do consentimento livre e esclarecido Obtido de todos os participantes antes da coleta dos dados.

## Introdução

Os agentes de combate às endemias são fundamentais na vigilância e no controle de doenças no Brasil e atuam nos territórios para identificar e reduzir riscos à saúde. Suas funções incluem pesquisa entomológica e malacológica, manejo ambiental, monitoramento de fatores ambientais e ações de educação e de mobilização comunitária. Além do domínio técnico em controle vetorial, os agentes de combate às endemias precisam atuar em integração com a atenção primária à saúde e compreender os determinantes sociais da saúde (1).

A atuação dos agentes de combate às endemias enfrenta desafios estruturais significativos. A fragmentação das ações de vigilância resulta na desarticulação entre setores, serviços e profissionais, o que geram práticas isoladas e pouco coordenadas. Isso compromete a efetividade das ações devido à dificuldade de integração com a atenção primária à saúde, à indefinição ou sobreposição de atribuições e à ausência de fluxos de comunicação eficazes para o planejamento conjunto (2-5). 

A formação dos agentes de combate às endemias tem se concentrado em capacitações operacionais específicas, sobretudo na identificação e na eliminação dos criadouros de vetores de doenças como dengue, malária e leishmaniose (3,6). Embora essa abordagem tenha contribuído para a redução de surtos, seu escopo restrito limita a atuação dos agentes de combate às endemias em estratégias mais amplas de saúde coletiva (5). A falta de integração efetiva entre os agentes de combate às endemias e as equipes da atenção primária à saúde compromete a eficácia das intervenções em saúde pública.

O compartilhamento de atribuições entre agentes de combate às endemias e agentes comunitários de saúde foi instituído na política nacional de atenção básica de 2017 (7), reforçado pelo guia técnico que orienta a integração entre vigilância em saúde e atenção primária à saúde (8). Entre as funções compartilhadas destacam-se o desenvolvimento de atividades de promoção da saúde e de prevenção de doenças e agravos, assim como a orientação da comunidade sobre sintomas, riscos e agentes transmissores. Os agentes também têm o papel fundamental de informar e de mobilizar a comunidade para implementar medidas simples de manejo ambiental e outras intervenções no ambiente para além do controle de vetores (7,8).

No âmbito do fortalecimento e da qualificação do sistema único de saúde foi instituído, por meio da Portaria GM/MS nº 3.241/2020, o Programa Saúde com Agente, destinado à formação especializada de agentes de combate às endemias e de agentes comunitários de saúde (9). Foi o primeiro curso técnico ofertado pelo Ministério da Saúde com vagas distribuídas em todo o país, alinhado à política nacional de atenção básica e, mais recentemente, à diretriz nacional de integração dessas duas categorias profissionais (7,1). 

O Programa Saúde com Agente é estruturado em atividades teóricas no ambiente virtual de aprendizagem e em práticas supervisionadas nos territórios, o que promove a articulação entre o conhecimento técnico e a realidade local. A etapa teórica é dividida em duas partes: a primeira, com temas comuns aos agentes de combate às endemias e aos agentes comunitários de saúde; e a segunda, dedicada a conhecimentos específicos de cada área. Essa formação incentiva o desenvolvimento de competências essenciais em vigilância ambiental, epidemiológica e sanitária (10).

Considerando o contexto do Programa Saúde com Agente, este estudo descritivo teve como objetivo apresentar a autopercepção dos conhecimentos e das habilidades dos agentes de combate às endemias antes do início da formação técnica específica. O estudo contribui para identificar lacunas no conhecimento e fornecer subsídios para o aprimoramento da qualificação profissional, alinhado às diretrizes da educação continuada em saúde. Os resultados poderão orientar estratégias educacionais mais eficazes e contextualizadas, o que fortalece as políticas públicas de vigilância em saúde no Brasil.

## Método

### Desenho do estudo

Este estudo é integrante do projeto de pesquisa intitulado “A formação no Programa Saúde com Agente e seu impacto na saúde das comunidades”. O objetivo foi descrever as habilidades autopercebidas dos estudantes do curso técnico em vigilância em saúde, antes da formação específica destinada exclusivamente aos agentes de combate às endemias, com vistas a identificar lacunas formativas para a qualificação técnica dessas categorias profissionais e subsidiar ajustes curriculares. 

### Contexto

O estudo foi realizado no âmbito do Programa Saúde com Agente, uma iniciativa do Ministério da Saúde destinada à formação técnica dos agentes de combate às endemias e dos agentes comunitários de saúde em todo o território nacional. O curso é estruturado em duas etapas: uma primeira, comum a ambas as categorias; e uma segunda, específica para cada uma delas. 

### Participantes

Foram incluídos neste estudo exclusivamente os agentes de combate às endemias matriculados no curso técnico em vigilância em saúde com ênfase no combate às endemias (42.976 indivíduos). A adesão dos participantes foi voluntária e precedida pela aceitação do termo de consentimento livre e esclarecido em formato digital, cuja concordância foi condição indispensável para acesso ao questionário sobre autopercepção de habilidades relacionadas ao trabalho como agente de combate à endemias.

A coleta de dados ocorreu por meio de um questionário online autopreenchido, enviado por e-mail aos estudantes no sexto mês do curso, com o período de resposta compreendido entre 8/2/2023 e 28/3/2023.

### Variáveis

Foram avaliadas variáveis sociodemográficas: sexo (feminino, masculino e prefiro não informar/outro), raça/cor da pele (categorizada conforme o Instituto Brasileiro de Geografica e Estatística em: parda, preta, branca, amarela, indígena e prefiro não informar), idade (em anos, categorizada em: 20-29, 30-39, 40-49, 50-59 e ≥60 anos), escolaridade (ensino médio incompleto, ensino médio completo, ensino superior incompleto, ensino superior completo, pós-graduação incompleta e pós-graduação completa), macrorregiões (Nordeste, Sudeste, Norte, Centro-Oeste e Sul), local de trabalho (urbano, rural, ribeirinho, indígena e outro), tempo de atuação como agentes de combate às endemias (em anos, categorizado em: 0-2, 3-5, 6-10, 11-15, 16-20 e >20 anos) e vínculo de trabalho (contrato direto com o município, contrato direto com o estado, contrato terceirizado, não sei/prefiro não informar e outro). 

As habilidades dos agentes de combate às endemias foram investigadas através de quatro domínios principais do questionário de autopercepção de habilidades: (a) educação, planejamento e ações no território, (b) vigilância ambiental, (c) vigilância epidemiológica, e (d) vigilância sanitária, subdivididos em duas dimensões: (1) reconhecimento de necessidades e planejamento de ações e (2) execução das atividades. Foram utilizados um total de 25 questões do questionário de autopercepção de habidades com escala Likert de cinco níveis (“não sei fazer”, “sei fazer um pouco”, “tenho dúvida”, “sei fazer”, “sei fazer muito bem”). 

### Fontes de dados e mensuração

Os dados foram coletados por meio de um questionário estruturado, elaborado especificamente para esta pesquisa, composto por duas seções principais: perfil sociodemográfico e autopercepção das habilidades dos agentes de combate às endemias. O instrumento não passou por um processo completo de validação psicométrica, porém, sua construção seguiu uma metodologia rigorosa, dividida em quatro etapas, descritas a seguir:

Etapa 1: revisão bibliográfica realizada em outubro de 2022 na Biblioteca Virtual em Saúde, com a inclusão de publicações entre 2018 e 2022 e de documentos como projetos pedagógicos, diretrizes da política nacional de atenção básica e legislações pertinentes às atribuições profissionais dos agentes de combate às endemias.

Etapa 2: construção do instrumento, organizado em seis domínios: educação, planejamento e ações no território, processo de trabalho, vigilância ambiental no território, vigilância epidemiológica no território, vigilância sanitária no território e primeiros socorros. Eles abrangem as competências técnicas necessárias, compreendidas nesta pesquisa como manifestação do “saber fazer”, correspondente à capacidade de mobilizar recursos cognitivos, socioafetivos e psicomotores para enfrentar situações complexas (11). Cada habilidade foi avaliada segundo duas dimensões, denominadas “reconhecimento de necessidades e planejamento de ações” e “execução”, mediante escala Likert de cinco níveis: “não sei fazer”, “sei fazer um pouco”, “tenho dúvida”, “sei fazer” e “sei fazer muito bem”.

Etapa 3: validação de conteúdo e estrutura semântica realizada por oito especialistas com doutorado e experiência na área, conforme recomendação metodológica de Lynn, que estipula participação ideal entre cinco e dez especialistas.

Etapa 4: validação aparente (*face validity*) realizada com 15 profissionais, selecionados aleatoriamente, entre agentes de combate às endemias e agentes comunitários de saúde. Todos possuíam experiência mínima de seis meses na função e na atuação ativa. O objetivo foi avaliar a clareza, a compreensão, a relevância e colher sugestões de aprimoramento do questionário. 

O instrumento final contou com 47 questões distribuídas em seis domínios. Para este estudo, foram selecionadas 25 questões pertencentes a quatro deles (descritas na subseção “Variáveis”).

### Viés

Devido à coleta online e à natureza autopreenchida do questionário, podem existir vieses de seleção e de informação, os quais são discutidos na seção Discussão, ao discorrer sobre as limitações do estudo. Foram adotadas medidas para minimizar vieses, como exclusão de respostas duplicadas e recusas.

### Tamanho do estudo 

Dos alunos matriculados que acessaram o questionário no período disponível, 51 optaram por não participar da pesquisa, 588 respostas duplicadas foram identificadas e excluídas, e 440 participantes não responderam integralmente às questões relativas às habilidades avaliadas. A amostra final contou com 3.730 participantes. Não foi realizada uma amostragem probabilística e configurou-se uma amostra por conveniência dentro da população elegível.

### Variáveis quantitativas

As variáveis quantitativas contínuas, como idade e tempo de atuação, foram categorizadas em faixas pré-definidas para facilitar a análise e minimizar possíveis distorções causadas por valores extremos. As categorias foram estabelecidas com base em intervalos relevantes para a caracterização dos participantes, além de serem compatíveis com a literatura disponível.

### Métodos estatísticos

A organização e a limpeza dos dados foram realizadas no Microsoft Excel. As análises estatísticas foram conduzidas utilizando o software R, versão 4.4.1. Foram calculadas frequências absolutas e relativas para as variáveis categóricas e para os domínios e as dimensões das habilidades avaliadas. Consideraram-se válidas somente as respostas completas. 

## Resultados

Dos 3.730 agentes de combate às endemias participantes, 51,8% eram do sexo feminino. O grupo de raça/cor da pele negra (pretos e pardos) predominou, o que correspondeu a 67,0% da amostra. 49,1%, quase metade da amostra, possuía ensino superior completo ou incompleto, inclusive níveis de pós-graduação. 90,6% trabalhava em áreas urbanas e 39,7% se concentrava na região Nordeste. 67,9% tinham vínculo empregatício direto com o município. A faixa etária predominante, 39,9%, foi 40-49 anos. 25,7% tinham tempo de atuação no trabalho entre 11-15 anos (Tabela 1).

**Tabela 1:** Características sociodemográficas da amostra de agentes de combate às endemias participantes do Programa Saúde com Agente. Brasil, 2023 (n=3.730)

Características	n (%)
**Sexo **	
Feminino	1.934 (51,8)
Masculino	1.778 (47,7)
Prefiro não informar/Outro	18 (0,5)
**Raça/cor da pele**	
Parda	1.986 (53,2)
Branca	1.128 (30,2)
Preta	514 (13,8)
Amarela	44 (1,2)
Indígena	31 (0,8)
Prefiro não informar	27 (0,7)
**Idade **(anos)	
20-29	243 (6,5)
30-39	1.161 (31,1)
40-49	1.489 (39,9)
50-59	693 (18,6)
≥60	144 (3,9)
Escolaridade	
Ensino médio incompleto	50 (1,3)
Ensino médio completo	1.852 (49,7)
Ensino superior incompleto	522 (14,0)
Ensino superior completo	808 (21,7)
Ensino de pós-graduação incompleto	93 (2,5)
Ensino de pós-graduação completo	405 (10,9)
Macrorregiões	
Nordeste	1.480 (39,7)
Sudeste	1.126 (30,2)
Norte	434 (11,6)
Centro-Oeste	346 (9,3)
Sul	344 (9,2)
**Local de trabalho**	
Urbano	3.378 (90,6)
Rural	243 (6,5)
Área ribeirinha	15 (0,4)
Área indígena	8 (0,2)
Outro	86 (2,3)
**Tempo de trabalho **(anos)	
0-2	374 (10,0)
3-5	477 (12,8)
6-10	807 (21,6)
11-15	959 (25,7)
16-20	604 (16,2)
>20	509 (13,7)
**Vínculo de trabalho **	
Contrato direto com o município	2.533 (67,9)
Contrato direto com o estado	100 (2,7)
Contrato terceirizado (empresa, fundação ou organização social)	48 (1,3)
Não sei/Prefiro não informar	113 (3,0)
Outro	936 (25,1)

Entre 79,0% e 94,0% dos agentes de combate às endemias referiram “saber fazer” ou “saber fazer muito bem” as atividades propostas, com os maiores percentuais observados em ações educativas sobre prevenção de doenças endêmicas, 90,3%, controle de vetores, 94,0%, e aplicação de agrotóxicos, 90,5% (Tabelas 2, 4 e 5).

**Tabela 2:** Habilidades de educação, planejamento e ações no território, antes da formação técnica específica aos agentes de combate às endemias no Programa Saúde com Agente. Brasil, 2023 (n=3.730)

Educação, planejamento e ações no território	Não sei fazer n (%)	Sei fazer um pouco n (%)	Tenho dúvida n (%)	Sei fazer n (%)	Sei fazer muito bem n (%)	
**Reconhecimento de necessidades e planejamento de ações**						
Planejar junto à comunidade ações de educação para a promoção da saúde.	52 (1,4)	569 (15,3)	171 (4,6)	2.116 (56,7)	822 (22,0)	
Planejar junto à comunidade ações de combate aos danos ambientais.	112 (3,0)	643 (17,2)	436 (11,7)	1.929 (51,7)	610 (16,4)	
Identificar situações de risco social no território (situações de violência, vulnerabilidade social entre outras).	56 (1,5)	406 (10,9)	308 (8,2)	2085 (55,9)	875 (23,5)	
Execução						
Mobilizar a comunidade para participar de ações educativas.	60 (1,6)	416 (11,1)	178 (4,8)	2.058 (55,2)	1.018 (27,3)	
Realizar ações de educação sobre prevenção de doenças endêmicas.	26 (0,7)	235 (6,3)	101 (2,7)	1.949 (52,3)	1.419 (38,0)	
Realizar ações de combate aos danos ambientais.	109 (2,9)	574 (15,4)	448 (12,0)	1.890 (50,7)	709 (19,0)	

As atividades de vigilância ambiental apresentaram os maiores percentuais de agentes que declararam não saber realizar as ações, com variação de 9,5% a 13,8% nas atividades relacionadas a danos causados por chuvas intensas, períodos de seca, qualidade do ar e aproveitamento de rejeitos orgânicos (Tabela 3). Quando incluímos os agentes que relataram saber fazer pouco ou ter dúvidas, o percentual alcançou 53,0% para essas atividades (Tabela 3). 

**Tabela 3:** Habilidades de vigilância ambiental no território, antes da formação técnica dos agentes de combate às endemias no Programa Saúde com Agente. Brasil, 2023 (n=3.730)

Vigilância ambiental no território	Não sei fazer n (%)	Sei fazer um pouco n (%)	Tenho dúvida n (%)	Sei fazer n (%)	Sei fazer muito bem n (%)
**Reconhecimento de necessidades e planejamento de ações**					
Planejar ações de combate aos danos causados ao ambiente (pelas secas, pelas chuvas intensas e enchentes entre outros).	514 (13,8)	748 (20,1)	713 (19,1)	1.326 (35,5)	429 (11,5)
Planejar ações para recolher copos, garrafas, vidros e plásticos para reciclagem.	70 (1,9)	318 (8,5)	126 (3,4)	1.866 (50,0)	1.350 (36,2)
Planejar ações para aproveitamento do lixo e realização de compostagem.	446 (11,9)	716 (19,2)	629 (16,9)	1.429 (38,3)	510 (13,7)
Execução					
Realizar ações com a comunidade para diminuir a poluição ambiental (do ar, das águas e sonoras)	353 (9,5)	727 (19,5)	530 (14,2)	1.632 (43,7)	488 (13,1)
Realizar ações com a comunidade para diminuir os danos ao ambiente provocados por secas, chuvas intensas e enchentes entre outros.	415 (11,1)	735 (19,7)	668 (17,9)	1.481 (39,7)	431 (11,6)
Realizar ações com a comunidade para recolher copos, garrafas, vidros e plásticos para reciclagem.	100 (2,7)	402 (10,8)	175 (4,7)	1.935 (51,9)	1.118 (29,9)
Realizar ações junto com a comunidade para aproveitamento do lixo orgânico e realização de compostagem.	441 (11,8)	714 (19,1)	645 (17,3)	1.423 (38,2)	507 (13,6)

Habilidades de vigilância epidemiológica e de planejamento junto à comunidade apresentaram altos índices de autopercepção de competência, 79,1% para registros de ações e 94,0% para controle de doenças transmitidas por vetores. Indicadores relacionados à saúde do trabalhador situaram-se entre 58,8% e 61,4% para os que declaram saber fazer (Tabela 4). 

**Tabela 4:** Habilidades de vigilância epidemiológica no território, antes da formação técnica dos agentes de combate às endemias no Programa Saúde com Agente. Brasil, 2023 (n=3.730)

Vigilância epidemiológica no território	Não sei fazer n (%)	Sei fazer um pouco n (%)	Tenho dúvida n (%)	Sei fazer n (%)	Sei fazer muito bem n (%)
**Reconhecimento de necessidades e planejamento de ações**					
Planejar junto à comunidade ações de prevenção de doenças endêmicas.	47 (1,3)	350 (9,4)	150 (4,0)	1.950 (52,3)	1.233 (33,0)
Planejar ações de promoção da saúde do trabalhador e prevenção de doenças relacionadas ao trabalho.	259 (6,9)	618 (16,6)	565 (15,1)	1.658 (44,5)	630 (16,9)
Planejar ações que acolham as necessidades e as demandas da comunidade.	207 (5,6)	697 (18,7)	498 (13,3)	1.736 (46,5)	592 (15,9)
Execução					
Estimular ações de promoção da saúde do trabalhador e prevenção de doenças relacionadas ao trabalho.	299 (8,0)	654 (17,5)	585 (15,7)	1.591 (42,7)	601 (16,1)
Realizar ações para o controle de doenças transmitidas por vetores (dengue, zika, chikungunya, febre amarela entre outras).	19 (0,5)	145 (3,9)	60 (1,6)	1.520 (40,8)	1.986 (53,2)
Registrar os dados das ações de vigilância epidemiológica desenvolvidas.	143 (3,8)	332 (8,9)	305 (8,2)	1.678 (45,0)	1.272 (34,1)

A vigilância sanitária mostrou altas taxas de autopercepção de domínio para identificação e orientação sobre zoonoses, 84,8%, para vistorias sanitárias, 82,3%, e para aplicação de agrotóxicos, 90,5%. Em relação à destinação inadequada do lixo, aos riscos de desabamento e ao consumo de produtos inadequados, entre 59,9% e 67,5% dos agentes de combate às endemias relataram domínio das práticas avaliadas. Entre 2,5% e 7,8% declararam ausência de competência nas atividades (Tabela 5).

**Tabela 5:** Habilidades de vigilância sanitária no território, antes da formação técnica dos agentes de combate às endemias no Programa Saúde com Agente. Brasil, 2023 (n=3.730)

Vigilância sanitária no território	Não sei fazer n (%)	Sei fazer um pouco n (%)	Tenho dúvida n (%)	Sei fazer n (%)	Sei fazer muito bem n (%)
**Reconhecimento de necessidades e planejamento de ações**					
Identificar problemas no território (destino inadequado do lixo, riscos de desabamento de encostas entre outros).	270 (7,2)	567 (15,2)	375 (10,1)	1.730 (46,4)	788 (21,1)
Identificar riscos para transmissão de doenças por animais.	93 (2,5)	535 (14,3)	286 (7,7)	1.892 (50,7)	924 (24,8)
Planejar ações que evitem o consumo de produtos inadequados (alimentos, água entre outros) pela comunidade.	292 (7,8)	649 (17,4)	555 (14,9)	1.696 (45,5)	538 (14,4)
Execução					
Realizar vistorias sanitárias em domicílios terrenos baldios, depósitos e estabelecimentos comerciais.	149 (4,0)	283 (7,6)	229 (6,1)	1.528 (41,0)	1.541 (41,3)
Orientar a comunidade para os cuidados com animais (cães, gatos, cavalos entre outros) para evitar transmissão de doenças.	70 (1,9)	323 (8,7)	171 (4,6)	1.799 (48,2)	1.367 (36,6)
Aplicar agrotóxicos (larvicidas e inseticidas).	83 (2,2)	149 (4,0)	122 (3,3)	1.511 (40,5)	1.865 (50,0)

## Discussão

A maior parte dos agentes de combate às endemias investigados neste estudo relatou elevada confiança nas atividades de prevenção e de controle de doenças endêmicas, vigilância epidemiológica e manuseio de agrotóxicos. Identificaram-se lacunas, porém, em relação à vigilância ambiental, especialmente no manejo de danos decorrentes de eventos climáticos e no aproveitamento de rejeitos orgânicos. 

A metodologia baseada em questionários autorreferidos, embora sujeita a viés de superestimação de competências, revelou-se confiável na identificação de áreas de domínio e de fragilidades, pois a consistência interna dos achados sustentou os indicadores de excelência e as dificuldades apontadas (12). 

Apesar de a amostra ser composta por alunos de curso técnico com aulas remotas, a coleta online pode ter restringido indivíduos com menor acesso e fluência digital, o que leva a sub-representação de segmentos mais vulneráveis ou com menor escolaridade (13). 

O estudo avançou em termos demográficos, levando-se em consideração que a literatura predominantemente é limitada a estudos regionais de pequeno porte (14,15). 

Embora este estudo não tenha incluído avaliação objetiva das habilidades, reconhece-se que cada investigação científica oferece uma perspectiva específica do fenômeno estudado, o que contribuiu com elementos particulares para a compreensão mais ampla do tema. Os achados aqui apresentados forneceram subsídios importantes sobre a autopercepção dos agentes de combate às endemias.

Observou-se um aumento importante na participação feminina na profissão, historicamente dominada por homens, o que corroborou achados de estudos anteriores e tendências em outras ocupações da saúde (14,15). A predominância etária acima dos 40 anos, combinada ao tempo médio de serviço superior a 10 anos, sugeriu uma equipe experiente, fator que pode favorecer a qualidade da prática profissional (5,14,15). Reforça-se a necessidade contínua de estratégias de educação permanente, alinhadas à política nacional de educação permanente em saúde, para atualizar conhecimentos e práticas diante dos avanços tecnológicos e das demandas emergentes (16-18). 

A maioria dos agentes de combate às endemias se autodeclarou de raça/cor da pele parda, o que compôs, juntamente com os que se autodeclaram de raça/cor da pele preta, o contingente de pessoas negras na categoria profissional. Essa composição racial é consistente com estudos anteriores sobre o perfil sociodemográfico dos agentes de combate às endemias e dos agentes comunitários de saúde no Brasil, o que reflete a inserção predominante de pessoas negras especialmente em funções de base comunitária (19). 

A escolaridade predominante neste estudo foi compatível com os requisitos mínimos para o cargo e um contingente significativo apresentou escolaridade superior à exigida, o que indicou potencial para qualificação e para ampliação das competências técnicas na categoria (14,15,19). Ressalta-se a importância de políticas de formação continuada e de valorização profissional que considerem a heterogeneidade educacional e estimulem o desenvolvimento de habilidades críticas e intersetoriais (1,19).

Os agentes de combate às endemias relataram, neste estudo, domínio da maioria das atividades. Os menores domínios percebidos foram na vigilância ambiental, especialmente em temas como danos ambientais causados por chuvas intensas, secas, qualidade do ar e aproveitamento de rejeitos orgânicos. A autopercepção de menor proficiência, inclusive, no campo da educação foi observada no planejamento e na execução de atividades ambientais. Essa lacuna pode advir da ênfase histórica em treinamentos operacionais curtos e informais e de desafios estruturais no sistema único de saúde, como falta de clareza nas atribuições dos agentes de combate às endemias e dificuldades na operacionalização de ações intersetoriais (20-23). 

A fragmentação entre os agentes de combate às endemias e os agentes comunitários de saúde agrava essa limitação, o que compromete a efetividade das ações de vigilância e a adesão comunitária. Entraves organizacionais e processuais, como a falta de fluxos de comunicação ou protocolos integrados, também prejudicam a articulação entre atenção primária e vigilância em saúde (2,24,25). Superar essa fragmentação exige a articulação de competências em modelos integrados de trabalho, que combinam funções generalistas e especializadas, o que fortalece o vínculo territorial e a resposta a determinantes sociais e ambientais da saúde.

A relação entre saúde e meio ambiente é reconhecida desde 1986, a partir da 8ª Conferência Nacional de Saúde, e foi consolidada na Constituição de 1988 e na Lei nº 8.080/1990 (26,27), que definem a vigilância ambiental como parte fundamental do Sistema Único de Saúde. O controle de endemias, mesmo assim, concentrou-se historicamente em ações pontuais, sem considerar de forma abrangente os determinantes sociais e ambientais da saúde (23). A reorganização da secretaria de vigilância em saúde e ambiente (Decreto nº 11.358/2023) (28) e a incorporação dos agentes de combate às endemias à atenção primária à saúde pela política nacional de atenção básica, de 2017, representam marcos para ampliar as atribuições desses profissionais em vigilância ambiental e em saúde do trabalhador (7).

Este estudo mostrou que os percentuais mais altos de autopercepção concentraram-se em atividades de controle vetorial, em ações educativas e em manejo de agrotóxicos, o que refletiu o modelo sanitarista campanhista de atuação reativa (4). Tal dinâmica foi observada no controle da dengue e em investigações sobre hanseníase e doença de Chagas (29,30), o que sugeriu um padrão sistêmico de atuação compartimentalizada. Para superar essa compartimentalização, é essencial capacitar os agentes de combate às endemias em planejamento e em execução de ações intersetoriais, o que leva à valorização de práticas integradas no território. 

Reforça-se a necessidade de intensificar a articulação entre atenção básica e vigilância em saúde por meio de iniciativas como o Programa Saúde com Agente, com vistas a um Sistema Único de Saúde mais resolutivo e participativo. Essa estratégia contribui para a transição do modelo tradicional de “combate de endemias”, com uso de agrotóxicos, para um modelo de “controle de endemias”, pautado em ações territoriais e em educação ambiental da população, o que demanda um processo de mudança estrutural de longo prazo (1,15,20). 

Este estudo ofereceu um diagnóstico situacional antes da formação técnica direcionada aos agentes de combate às endemias no Programa Saúde com Agente, com destaque para competências consolidadas e necessidades formativas específicas. Esses achados podem orientar o desenvolvimento curricular e subsidiar estratégias pedagógicas direcionadas às lacunas identificadas, o que contribui para uma formação integrada e qualificada no âmbito do Sistema Único de Saúde.

## Data Availability

Os dados fazem parte de pesquisa em andamento e serão disponibilizados futuramente pelo Ministério da Saúde, em formato anonimizado.
